# The effects of a novel bicarbonate loading protocol on serum bicarbonate concentration: a randomized controlled trial

**DOI:** 10.1186/s12970-019-0309-4

**Published:** 2019-09-18

**Authors:** Adam Marcus, Amerigo Rossi, Andrew Cornwell, Steven A. Hawkins, Nazareth Khodiguian

**Affiliations:** 1 Division of Athletic Training, Health and Exercise Science, Long Island University Brooklyn, Brooklyn, NY 11201 USA; 2 School of Kinesiology and Nutritional Science, California State University Los Angeles, 5151 State University Boulevard, Los Angeles, CA USA; 3 Department of Exercise Science, California Lutheran University, 60 West Olsen Road, Thousand Oaks, CA USA

**Keywords:** Ergogenic, Running, Elite, Bicarbonate, Lactate, Buffer, Nutrition, Supplement

## Abstract

**Background:**

Previous studies have shown that sodium bicarbonate ingestion may enhance intense exercise performance, but may also cause severe gastrointestinal distress. The purpose of this study was to determine whether a modified sodium bicarbonate (SB) ingestion protocol would elevate serum bicarbonate concentration more than previous methods without causing gastrointestinal distress.

**Methods:**

In randomized order, seven (5 men, 2 women) elite middle-distance runners ingested either placebo, Modified SB (600 mg·kg^− 1^ over 19.5 h), or Acute SB (300 mg·kg^− 1^) in opaque gelatin capsules. Baseline and post-ingestion blood samples were analyzed for bicarbonate, pH, sodium, hematocrit, and lactate. Repeated measures ANOVA (2 time points × 3 conditions) were analyzed to determine differences in serum bicarbonate, lactate, sodium, blood pH, and hematocrit. Gastrointestinal distress was assessed via self-report on a Likert scale of 1–10. Simple (condition) and repeated (time) within-participant contrasts were used to determine the location of any statistically significant main and interaction effects (*p* ≤ 0.05).

**Results:**

Both Modified SB (7.6 mmol·L^− 1^, *p* < 0.01) and Acute SB (5.8 mmol·L^− 1^, *p* < 0.01) increased serum bicarbonate concentration compared to the placebo (*p* ≤ 0.05). Post-ingestion serum bicarbonate concentration was significantly higher for the Modified SB (34.7 ± 2.2 mmol·L^− 1^, 28.0% increase) trials than the Acute SB (33.5 ± 2.0 mmol·L^− 1^, 20.9% increase) trials (*p* = 0.05). There was no reported severe GI distress in the Modified SB trials, but two cases in the Acute SB trials.

**Conclusions:**

Modified SB elevated serum bicarbonate concentration more than Acute SB, without any severe gastrointestinal side effects. Consequently, it is recommended that future experimentation involving SB by researchers and athletes use the novel ingestion protocol described in this study due to its potential for improved effectiveness and reduced gastrointestinal impact.

**Trial registration:**

*ClinicalTrials.gov* *,* NCT03813329 *. Registered 23 January 2019 - Retrospectively registered,*

## Introduction

Sodium bicarbonate has been shown to increase blood alkalosis and bicarbonate levels, as well as exercise capacity [1–3]. Several studies have investigated the effect of sodium bicarbonate (SB) ingestion on exercise performance [4], with mostly equivocal findings. One systematic review had found that SB ingestion was one of the most effective ergogenic aids for middle distance running [5], whereas a more recent systematic review found that sodium bicarbonate was found to be an effective ergogenic aid in 11 out of 20 randomized controlled trials of performance lasting less than 4 minutes [6]. Considering that the difference between 1st and 12th place in the most recent men’s Olympic 1500 m race in Athletics (2016) was only 1.73 s (0.75% of the total time), and sodium bicarbonate may improve high-intensity running performance in trained athletes by 2–3% [7], investigating optimal SB ingestion protocols may be of high importance.

The discrepancy in previous findings may be due to side effects of typical dosing strategies. Most studies of the effects of SB on performance have administered a standard acute dosages of sodium bicarbonate (300 mg·kg^− 1^ body weight), which may cause gastrointestinal (GI) distress [8–10] and potentially minimize any ergogenic effect of the bicarbonate ingestion [11]. For example, Saunders et al. [12] observed improvements in high-intensity cycling, but only in those that did not experience GI distress. Larger acute doses of SB (i.e. 500 mg·kg^− 1^) elevate serum bicarbonate concentration to a greater extent [11, 13], indicating a dose response [13], but may also cause more severe GI distress [14].

Because higher serum SB may have a greater ergogenic effect on performance, but larger doses induce greater GI distress, several studies have attempted to administer relatively small doses over several days [15–17] in order to significantly elevate blood bicarbonate without inducing GI distress. While these protocols, ranging from 3 to 10 days, avoided causing GI distress, they only increased blood bicarbonate concentration approximately 10%, which is much less than what typically been found in acute ingestion protocols [9, 18, 19]. More research needs to be conducted to determine the most effective chronic dosing strategy [6].

The primary purpose of the present study, therefore, was to evaluate the effect of a novel 1-day SB ingestion protocol on serum bicarbonate and GI distress. It was hypothesized that this novel protocol would significantly elevate serum bicarbonate concentration and produce less GI distress than typical acute SB ingestion.

## Methods

### Study design

Blood samples were collected at baseline and following placebo (CaCO_3_), acute sodium bicarbonate (AcuteSB) ingestion, and modified sodium bicarbonate (ModSB) ingestion protocols to determine the effects on serum bicarbonate, sodium and lactate concentrations, as well as on serum pH and blood hematocrit. The study design was a randomized double-blind crossover. The order of ingestion protocols was counter-balanced using a Latin square to minimize the potential of a learning effect. All tests were administered between 8 and 11 AM, and were conducted at the same time of day for each participant. A period of 7–10 days was allowed between trials to ensure the ingested substances had returned to baseline prior to the subsequent trial [17]. Participants were instructed to maintain their normal training patterns throughout the study, and to refrain from intense training for at least 48 hours prior to each test. Participants were also instructed to eat the same high-carbohydrate breakfast approximately 3 hours prior to each post-ingestion assessment.

### Participants

Ten elite middle-distance runners (6 men, 4 women) from running teams in the Los Angeles area volunteered to participate. Inclusion criteria were: 1). Peak oxygen consumption greater than 60 ml·kg^− 1^·min^− 1^ (men) or 50 ml·kg^− 1^·min^− 1^ (women); 2). Currently training, defined as at least 5 days·week^− 1^ of running; 3). Elite-level performance (750 or more points on the International Associations of Athletics Federations Scoring Table) for an 800 m–5000 m race during the preceding 6 months. Elite athletes were selected because they are a sample population that would actually utilize sodium bicarbonate supplementation.

### Preliminary testing

Preliminary testing occurred at least 3 days prior to the 1st trial. Participants self-reported their training status and race results. Height and weight were assessed with a stadiometer. Body fat was assessed by hydrodensitometry [20], with residual lung volume measured by the oxygen dilution technique [21], in order to accurately assess body composition in this highly trained sample. After a brief rest, participants underwent maximal oxygen consumption (Vo_2peak_) testing using a modified treadmill protocol: Elevation was fixed at 8%; speed began at 4 mph and increased 1 mph every 2 minutes until volitional exhaustion. Based on pilot testing, this protocol was utilized so that a safe high-intensity treadmill running speed at 110% Vo_2peak_ could be calculated for elite runners. Minute ventilation, Vo_2_, and Vco_2_ were determined during the Vo_2peak_ test by a Vmax 29 metabolic cart (Sensormedics, Loma Linda, CA). Heart rate was assessed during the Vo_2peak_ test by electrocardiography using CM_5_ electrode configuration. Achievement of Vo_2peak_ was confirmed if at least two of the following criteria were met: 1). Respiratory exchange ratio greater than 1.05; 2). Heart rate within 10 bpm of age-predicted maximum; 3). Plateau in Vo_2_ with increasing workloads.

### Ingestion protocols

The ModSB protocol consisted of 4 progressively larger doses of SB administered with progressively shorter time intervals between doses consumed during the 19.5-h period prior to post-ingestion testing (Table 1). This protocol was developed in order to deliver a maximum amount of sodium bicarbonate in smaller individual doses that would not cause GI distress (≤ 200 mg·kg^− 1^), while maintaining short total consumption time to minimize sodium consumption. Calcium carbonate was chosen as the placebo because it had repeatedly been used successfully in previous sodium bicarbonate research [22]. The calcium carbonate ingestion protocol (placebo) simulated the ModSB protocol. In order to maintain the double-blind design of the study, the first three doses of the Acute SB protocol contained the placebo, and only the last dose contained 300 mg·kg^− 1^ of SB.

**Table 1:** Timing and Dosages of the Three Ingestion Protocols

Time		Placebo	AcuteSB	ModSB
− 24 h	Baseline blood draw			
− 19.5 h	Dose 1	110 mg•kg^− 1^ CaCO_3_	110 mg•kg^− 1^ CaCO_3_	110 mg•kg^− 1^ NaHCO_3_
− 11.5 h	Dose 2	130 mg•kg^− 1^ CaCO_3_	130 mg•kg^− 1^ CaCO_3_	130 mg•kg^− 1^ NaHCO_3_
−4.5 h	Dose 3	160 mg•kg^− 1^ CaCO_3_	160 mg•kg^− 1^ CaCO_3_	160 mg•kg^− 1^ NaHCO_3_
- 1.5 h	Dose 4	200 mg•kg^− 1^ CaCO_3_	300 mg•kg^− 1^ NaHCO_3_	200 mg•kg^− 1^ NaHCO_3_
0	Post-ingestion blood draw			

AcuteSB: Acute sodium bicarbonate ingestion

ModSB: Modified sodium bicarbonate ingestion

For each trial, participants arrived at the lab 24 h prior to the post-ingestion test to have a baseline blood draw and receive 4 doses of the substance(s) to be ingested in numbered sealed bags with a detailed ingestion schedule and a 750 ml bottle of water. Beginning 19.5 h prior to the post-ingestion test, participants ingested each dose with 750 ml of water according to the ingestion schedule. Participants were asked to record the timing of each ingested dose for confirmation. The same number of capsules was used per dose across trials for each participant and doses were encased in opaque gelatin capsules (size “00”) to mask the flavor and granularity of the respective substances.

Participants self-reported their respective levels of GI distress on a Likert scale of 1–10 (1 = no GI distress, 10 = worst possible GI distress). The data were classified a priori as 1–3 equal to limited GI distress, 4–7 as moderate GI distress, and 8–10 as severe GI distress.

### Blood analysis

Ten ml of blood was drawn via forearm venipuncture twice for each condition. Baseline blood draws occurred 24 h prior to the post-ingestion test in order to minimize the effect of diurnal variations in hematocrit, plasma sodium and bicarbonate concentrations [23]. The post-ingestion blood draws were taken 78 ± 7 minutes following the 4th dose.

Two capillary tubes were immediately filled from the blood samples to be tested for hematocrit via the microhematocrit method [24] in order to evaluate changes in blood composition following the ingestion protocols. The remaining blood was allowed to coagulate at room temperature for 25 minutes and was then spun in a refrigerated centrifuge for 10 min. The serum was pipetted into three small vials, one of which was placed in a − 80 °C freezer for later analysis of serum sodium and lactate concentrations. The serum in one vial was immediately analyzed for pH (Orion 720A+, Thermo Electron Corporation, Waltham, MA) and the other was used to measure bicarbonate concentration in duplicate with a carbon dioxide liquid stable reagent method (TR28321, Thermo Electron Corporation, Waltham, MA) using a spectrophotometer (Lambda 20, PerkinElmer, Waltham, MA) [25]. Once all the participants had been tested, the frozen samples were thawed and analyzed for sodium (Vitros DT60 II, Ortho-Clinical Diagnostics, Rochester, NY) and lactate concentrations (Vitros DT60 II, Ortho-Clinical Diagnostics, Rochester, NY) [26].

### Statistical analysis

Means and standard deviations were calculated (X ± SD) for all measurements. Repeated measures ANOVA with factorial design (2 time points × 3 conditions) were analyzed to determine significant interactions between time (baseline, post-ingestion) and condition (Placebo, AcuteSB, ModSB) for blood parameters. Simple (condition) and repeated (time) within-participant contrasts were used to determine the location of any significant differences. Paired-sample t-tests were analyzed to determine differences in blood parameters at baseline. In order to evaluate differences following the ingestion of AcuteSB and ModSB, effect sizes were calculated for each variable. Because of the small sample size, corrected effect sizes were calculated using Hedge’s g. All data analyses were performed with SPSS 25.0. Statistical significance was established at *p* ≤ 0.05.

## Results

It was hypothesized that this novel protocol would significantly elevate serum bicarbonate concentration and produce less GI distress than typical acute SB ingestion. Serum bicarbonate and GI distress were primary outcomes. Hematocrit, pH, serum sodium, and serum lactate were secondary outcomes.

Ten participants enrolled in the study. Three participants dropped out for reasons unrelated to the study (2 participants were injured during training and 1 withdrew for undisclosed personal reasons) and were not included in the data analysis. Characteristics of the remaining seven participants (5 men, 2 women) are summarized in Table 2. There were no significant differences for any of the blood parameters at baseline between conditions.

**Table 2:** Participant Characteristics (*N* = 7; Mean ± SD)

Age (yrs)	Height (cm)	Weight (kg)	Body fat (%)	VO_2peak_ (ml·kg^− 1^·min^− 1^)	HR_max_ (bpm)	Running (km·wk.^− 1^)
28 ± 6	170 ± 8	61.5 ± 8.1	8.9 ± 4.6	70.8 ± 9.0	191 ± 5	81 ± 25

**Fig. 1:**
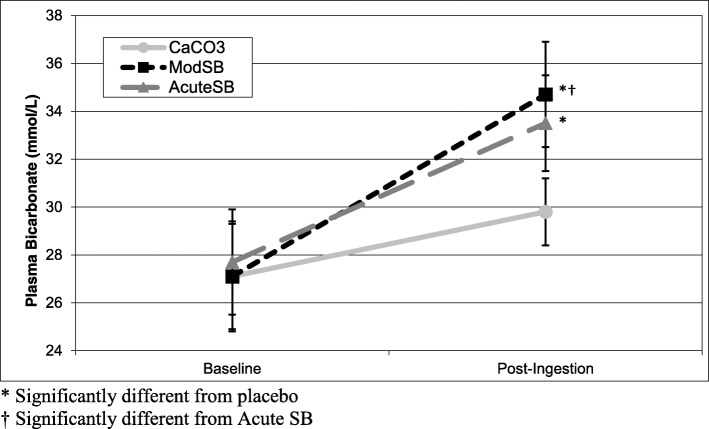
Serum Bicarbonate Concentration at Baseline, Post-ingestion. * Significantly different from placebo. † Significantly different from Acute SB

There was a significant interaction effect between condition and time (F = 9.52, *p* < 0.01) for serum bicarbonate concentration (Fig. 1). Although the placebo trials induced a small (2.7 mmol·L^− 1^) but significant (*p* < 0.01) increase in serum bicarbonate concentration, contrasts revealed that there were significantly greater increases in serum bicarbonate concentration for the AcuteSB (5.8 mmol·L^− 1^, *p* < 0.01) and ModSB (7.6 mmol·L^− 1^, *p* < 0.01) conditions compared to the placebo from Baseline to post-ingestion. Furthermore, post-ingestion serum bicarbonate concentration was significantly higher (*p* = 0.05) for the ModSB condition (34.7 ± 2.2 mmol·L^− 1^) than the AcuteSB condition (33.5 ± 2.0 mmol·L^− 1^).

Two of the seven participants reported severe (8 and 9 out of 10) gastrointestinal distress following the ingestion of the last dose during the Acute SB ingestion protocol, whereas none of the participants reported severe GI distress following the ModSB or placebo ingestion protocols.

There was a statistically significant interaction effect for pH (F = 6.03, *p* = 0.02) from baseline to post-ingestion. Analysis of the contrasts indicated that the results for pH paralleled those for serum bicarbonate concentration. There was a significantly greater increase in pH from baseline to post-ingestion in the AcuteSB (0.11 units, *p* = 0.04) and the ModSB (0.09 units, *p* < 0.01) trials compared to the placebo trials (0.04 units).

There was also a significant interaction effect for sodium (F = 5.54, *p* = 0.03). However, contrasts revealed that neither AcuteSB (1.4 mmol·L^− 1^, *p* = 0.10) nor ModSB (1.6 mmol·L^− 1^, *p* = 0.06) protocols significantly changed serum sodium concentrations compared to the placebo (− 1.6 mmol·L^− 1^).

There was a significant interaction effect between condition and time for hematocrit (F = 8.86, *p* < 0.01). Contrasts revealed that there were significant differences in the amount of change in hematocrit from baseline to post-ingestion between the ModSB (− 2.8 units, *p* < 0.01) and AcuteSB (− 1.6 units, *p* = 0.03) compared to the placebo (+ 0.6 units).

There was a significant main effect of time on lactate (*p* = 0.01), with an increase in all groups, but no interaction effect (*p* = 0.15).

There was a significant main effect of time on body weight (*p* = 0.020), with body weight increasing in all three groups slightly following the ingestion protocols. However, there were no significant interaction effects (*p* > 0.20).

Detailed values for each of the serum variables at each time point are summarized in Table 3, and individual participant values for serum bicarbonate are presented in Table 4.

**Table 3:** Mean (± standard deviation) Blood Hematocrit, and Serum Bicarbonate (HCO_3_^−^), pH, Sodium (Na^+^), and Lactate

	Placebo		AcuteSB			ModSB			
	Baseline	Post-ingestion	Baseline	Post-ingestion	*p*-value	Baseline	Post-ingestion	*p*-value	Effect size (Hedges’g)
HCO_3_^−^ (mmol·L^−1^)	27.1 (±2.3)	29.8 (±1.4)	27.7 (±1.3)	33.5 (±2.0)	< 0.01	27.1 (±2.2)	34.7^‡^ (±2.2)	< 0.01	0.53
pH	7.60 (±.04)	7.64 (±.05)	7.56 (±.06)	7.67 (±.08)	0.04	7.58 (±.05)	7.67 (±.04)	< 0.01	−0.18
Na^+^ (mmol·L^−1^)	138.8 (±1.3)	137.2 (±1.0)	139.7 (±2.3)	140.3 (±1.8)	0.10	138.8 (±1.3)	140.0 (±1.5)	0.06	0.32
Hematocrit (%)	45.9 (±3.5)	46.5 (±2.8)	46.8 (±2.2)	45.0 (±2.9)	0.03	46.2 (±3.5)	43.4 (±1.7)	< 0.01	−0.57
Lactate (mmol·L^−1^)	1.6 (±0.7)	1.7 (±0.2)	1.6 (±0.1)	2.0 (±0.3)	NS	1.4 (±0.2)	1.9 (±0.5)	NS	0.21

*p*-value indicates significantly different change from baseline compared to placebo (*p* ≤ 0.05)

Effect size reflects the difference in baseline to post-ingestion change between AcuteSB and ModSB

^‡^Significantly higher than AcuteSB (*p* ≤ 0.05)

AcuteSB: Acute sodium bicarbonate ingestion trial

ModSB: Modified sodium bicarbonate ingestion trial

NS: No significant interaction effect

**Table 4:** Individual serum bicarbonate values for each participant at baseline and post-ingestion

Participant #	Condition	Baseline (mmol·L^− 1^)	Post-ingestion (mmol·L^− 1^)	Change from Baseline (mmol·L^− 1^)	Change from Baseline (%)	Change Compared to Placebo (mmol·L^− 1^)
1	Placebo	26.4	29.4	3.0	11%	
	AcuteSB	30.0	35.6	5.7	19%	2.6
	ModSB	23.2	37.1	13.9	60%	10.9
2	Placebo	27.7	29.6	1.9	7%	
	AcuteSB	28.7	34.5	5.8	20%	3.9
	ModSB	28.2	34.7	6.4	23%	4.5
3	Placebo	30.9	32.3	1.4	5%	
	AcuteSB	28.3	30.8	2.5	9%	1.1
	ModSB	30.3	32.0	1.7	6%	0.3
4	Placebo	28.0	30.1	2.1	7%	
	AcuteSB	27.2	36.1	8.9	33%	6.8
	ModSB	28.3	35.0	6.7	24%	4.6
5	Placebo	23.1	27.7	4.6	20%	
	AcuteSB	26.8	33.5	6.7	25%	2.1
	ModSB	26.1	37.9	11.8	45%	7.2
6	Placebo	27.1	29.7	2.6	10%	
	AcuteSB	26.6	32.7	6.1	23%	3.5
	ModSB	26.6	34.4	7.8	29%	5.2
7	Placebo	26.8	29.5	2.8	10%	
	AcuteSB	26.6	31.3	4.7	18%	2.0
	ModSB	27.0	32.1	5.1	19%	2.3

*AcuteSB* Acute sodium bicarbonate ingestion trial

*ModSB* Modified sodium bicarbonate ingestion trial

## Discussion

The primary purpose of this study was to investigate if a novel sodium bicarbonate ingestion protocol (ModSB) would elevate serum bicarbonate concentration more effectively than previous protocols that have typically been employed (AcuteSB). We found that ModSB elevated serum bicarbonate concentration 30% more than AcuteSB, without any associated severe GI distress.

To the best of our knowledge, this was the first study to report a significantly greater increase in serum bicarbonate concentration compared to the changes elicited by typical sodium bicarbonate ingestion protocols. Previous studies have typically administered an acute dose (300 mg·kg^− 1^ of body weight) of sodium bicarbonate approximately 90 minutes prior to exercise, which has led to a ~ 20% elevation of serum bicarbonate concentration [9, 18, 19]. In the present study, a similar protocol, AcuteSB, elicited a similar elevation of serum bicarbonate concentration (20.9%). The ModSB ingestion protocol resulted in a significantly greater (28.0%) increase in serum bicarbonate concentration, supporting the hypothesis that oral administration of smaller but progressively incremental doses of sodium bicarbonate is more effective in increasing serum bicarbonate concentration.

Since each dose (110 mg·kg^− 1^ - 200 mg·kg^− 1^), was significantly lower than the dose known to cause GI distress (≥ 300 mg·kg^− 1^), ModSB trials did not induce severe GI distress in the present study. The AcuteSB ingestion, on the other hand, induced severe GI distress in two of the seven participants (29%). Other studies that have administered a 300 mg·kg^− 1^ dose also reported mild to severe GI distress in some participants [8, 9, 27], which may have had ergolytic effects.

In the present study calcium carbonate (CaCO_3_) was used as the placebo because of its widespread use as placebo in previous studies [9, 19]. However, the results of the present study indicated a 10% increase in serum bicarbonate concentration as a result of CaCO_3_ ingestion. Because the first three doses of the AcuteSB ingestion protocol contained progressively larger doses of the placebo (CaCO_3_), it is likely that some of the increase in serum bicarbonate concentration following AcuteSB trials actually resulted from the ingestion of placebo.

More sodium was ingested during the ModSB (~ 11,500 mg for 70 kg athlete) than the Acute SB trials (~ 5750 mg for 70 kg athlete), and none was consumed during the placebo trials. Correspondingly, there was a significant interaction effect and serum sodium concentration was increased, albeit insignificantly, in the two bicarbonate trials compared to the placebo. Hematocrit decreased significantly more in the two bicarbonate trials than in the placebo. Hematocrit and body mass can be used to estimate plasma volume [28]. The data from this study suggest that sodium bicarbonate ingestion and the associated increase in serum sodium concentration in the ModSB trial may have led to an increased plasma volume of approximately 6%. It has been shown that high sodium ingestion ~ 60–90 minutes prior to exercise increases plasma volume ~ 4% in men [29] and women [30]. However, since plasma volume was not directly assessed in this study, these findings should be interpreted with caution and investigated further.

There was also a significant main effect of time on weight, caused by an increase in weight across all conditions following the ingestion protocols of 0.4–0.9 kg. However, since there were no differences in body weight between conditions, the increase in weight was most likely due to the larger than normal total water consumption (3 L) during the ingestion protocol. Many participants noted that they were unaccustomed to consuming such large quantities of water.

One major strength of the current study is the use of only elite athletes in the protocol, increasing the generalizability of the findings to the intended population. The primary limitation, however, is the small sample size. Despite this limitation, the findings are strong enough to indicate the potential of a novel method for bicarbonate ingestion that is more beneficial and less detrimental that previous trials in elite athletes.

## Conclusions

The novel sodium bicarbonate ingestion protocol utilized in the present study elevated serum bicarbonate more than the conventional ingestion protocol without causing any associated severe gastrointestinal distress. To maximize the potential ergogenic effect and minimize the gastrointestinal side effects, athletes should try ingesting sodium bicarbonate according to the ingestion protocol utilized in this study.

## Data Availability

The datasets used and/or analysed during the current study are available from the corresponding author on reasonable request.

## Abbreviations

AcuteSB: Acute Sodium Bicarbonate
GI: Gastrointestinal
IRB: Institutional Review Board
ModSB: Modified Sodium Bicarbonate
SB: Sodium Bicarbonate
